# T4. IDENTIFICATION OF NEUROANATOMICAL SURROGATE MARKERS OF CHILDHOOD TRAUMA

**DOI:** 10.1093/schbul/sby016.280

**Published:** 2018-04-01

**Authors:** Theresa Haidl, Marlene Rosen, Mauro Seves, Thorsten Lichtenstein, Anne Ruef, Joseph Kambeitz, Nikolaos Koutsouleris

**Affiliations:** 1 University of Cologne; 2 University Hospital of Cologne; 3 Ludwig-Maximilian-University

## Abstract

**Background:**

Childhood trauma (CT) plays an important role in psychiatric disorders. It is associated with an increased risk for psychiatric disorders like major depression, anxiety disorders, dependency, post-traumatic stress disorders and even psychosis. There is a high incidence of CT in patients with psychosis, especially for physical and sexual abuse. Already in UHR-individuals increased CT could be observed. A study of Thompson and colleagues showed that 97% of their UHR sample reported a trauma in the past. 83% of the cases were physical abuse, 67% emotional abuse and 27% sexual abuse. Our aim was to investigate if there are neurobiological surrogate markers of trauma existing which can be detected by a multi pattern analysis.

**Methods:**

PRONIA (‘Personalized Prognostic Tools for Early Psychosis Management’) is a prospective collaboration project funded by the European Union under the 7th Framework Programme (grant agreement n° 602152). Considering a broad set of variables (sMRI, rsMRI, DTI, psychopathological, life event related and sociobiographic data, neurocognition, genomics and other blood derived parameters) as well as advanced statistical methods, PRONIA aims at developing an innovative multivariate prognostic tool enabling an individualized prediction of illness trajectories and outcome. Seven clinical centers in five European countries and in Australia participate in the evaluation of three clinical groups (subjects clinically at high risk of developing a psychosis [CHR], patients with a recent onset psychosis [ROP] and patients with a recent onset depression [ROD]) as well as healthy controls; planned sample size is n=1680. CT was assessed by the Childhood Trauma Questionnaire (CTQ). To identify neuroanatomical and functional surrogate markers of CT, a multi pattern analysis via Neurominer (NM) was conducted. An additional VBM analysis was performed to evaluate the results of the NM analysis.

**Results:**

We found that patients and HC could be separated very well by the CTQ pattern. Moreover, the classification among the patient groups yielded results that were not much better than a random classification. This finding underlines that CT is an overarching risk factor for mental diseases and not specific for single clinical entities. Furthermore, the decision scores that were conducted in the first NM analysis revealed highly significant negative correlations with grey matter changes in frontotemporal cortical areas, the anterior cingulate and the insular cortex. These areas are already discussed for CT in the literature.

**Discussion:**

CT seems to be a global risk factor for psychiatric disorders. Moreover, we could re-examine the results of our multi variate analysis successfully in a VBM procedure.

